# Quarantine Ships as Spaces of Bordering: The Securitization of Migration Policy in Italy During the COVID-19 Pandemic

**DOI:** 10.1177/01979183231154560

**Published:** 2023-03-02

**Authors:** Nicola Montagna

**Affiliations:** Middlesex University London

**Keywords:** COVID-19, floating hotspots, bordering

## Abstract

The COVID-19 pandemic presented an opportunity for bordering, that is, for measures that aim to delineate foreigners’ access to citizenship and membership and to further securitize migration policy. Across the globe, new border controls were introduced, stringent new international regulations applied, and hundreds of thousands of flights cancelled, all of which resulted in millions of travelers, including migrant workers and transnational commuters, being stranded. Among the areas affected by these bordering measures is the central Mediterranean migratory route to Italy. In Spring 2020, the Italian government introduced two measures aimed to block migrant arrivals by sea: the closure of ports to search-and-rescue (SAR) operations and the use of ships to quarantine migrants arriving on SAR ships. While the former was only partially implemented and then lifted in the summer of 2020, the latter has become a cornerstone of current securitization policies in Italy. This article — relying on semi-structured interviews with activists, non-governmental organization volunteers, human rights lawyers, and journalists — interrogates the use of quarantine ships during the pandemic as a means of stopping COVID-19's spread by irregular migrants arriving along the central Mediterranean. It shows how this measure, presented as a humanitarian mission to preserve public health, became an opportunity to securitize national and EU borders and how quarantine ships became spaces aimed at filtering and containing arriving migrants. The article aims to contribute to the debate around the bordering policy measures that characterize current EU migration governance and to consider their application during the pandemic.

## Introduction

A main consequence of the COVID-19 pandemic has been a reduction in mobility, with nearly all governments worldwide adopting restrictions on the internal and international movement of people in an attempt to contain the spread of the virus (Benton et al. 2021; Triandafyllidou 2020). As soon as the pandemic broke out, new border controls were introduced, more stringent international regulations applied, and hundreds of thousands of flights cancelled, resulting in millions of travelers, including migrant workers, transnational commuters, students, frequent flyers, and tourists, being stranded (ibid.). Although differing between countries (O’Brien and Eger 2020), these measures also affected the European Union (EU), with the total or partial closure of many transit locations, including ports, airports and land borders, increased controls and other restrictive measures at borders, and the de-facto temporary suspension of the Schengen Treaty — the mid-1980s agreement that established free movement for citizens and residents between the countries adhering to it (Benton et al. 2021; Sanchez and Achilli 2020).

Among the geographical areas affected by bordering and re-bordering measures is the central Mediterranean migratory route. In Spring 2020, the Italian government introduced two policy measures to block the arrival of non-EU migrants by sea from Libya and other countries of the southern Mediterranean coast: the closure of ports to search-and-rescue (SAR) operations and the use of ships to quarantine migrants arriving on SAR ships (Ambrosini 2021). While the former strategy was only partially implemented and then lifted in the summer of 2020, the latter has become a cornerstone of current border securitization policies in Italy. After limited initial use of this measure, by mid-September 2020, Italy had a fleet of seven quarantine ships chartered by Grandi Navi Veloci,^1^ run by the Italian Red Cross for health and logistic services, and moored outside critical landing areas, including Lampedusa, to guard its borders against the “peril” represented by the possible arrivals of COVID-19-positive migrants (Anderlini and Di Meo 2021). Within a short time, the scope of quarantine ships had widened to include not only migrants rescued at sea but also those who arrived by their own means and, for a short time period, migrants already settled in the reception system (Associazione per gli Studi Giuridici sull'Immigrazione 2021).

This article interrogates the use of quarantine ships in Italy as a means to stop COVID-19's spread by irregular migrants arriving along the central Mediterranean route. Its purpose is to examine how this measure became a securitarian bordering device, rather than a procedure aimed at protecting public health, and to contribute to the current debate on the bordering policy measures and practices that characterize current EU migration governance and consider their application during the pandemic. Through the use of primary sources including interviews with Red Cross volunteers and key informants from non-governmental organizations (NGOs), media, and grassroots associations, I show that quarantine ships were used as “floating hotspots,” creating a precedent for a previously rejected idea^2^ to take concrete shape. Similarly to so-called hotspots centers located in proximity to ports of arrival in the EU member-states in the Mediterranean designed by the 2015 European Agenda on Migration (European Commission 2015, 5), quarantine ships’ function was to identify and register arriving migrants, and, by doing so, operate a preventive and differential filtering (Giacomelli 2020; Queirolo and Rahola 2020; Tazzioli and Garelli 2018).

To develop this argument, the first section addresses the current debate on bordering and how bordering measures were enacted during the pandemic. The second section focuses on the research methods used for this article and discusses some issues arising from doing primary research on migration at a distance and during the pandemic. The third section analyzes the legal framework underlying the policy measure of quarantine ships in Italy. The fourth section investigates some aspects of Red Cross involvement in managing quarantine ships and the securitarian displacement of humanitarian aid as part of the bordering process. The fifth section focuses on quarantine's impact on migrants, and the final section returns to the notion of quarantine ships as “floating hotspots” and bordering spaces.

## Bordering, Security, and the Pandemic

Bordering has been increasingly debated in migration and border studies. The term designates social, relational, and symbolic processes and forms of spatial delimitations that take place at several levels and in a variety of contexts (Yuval-Davis, Wemyss and Cassidy 2019). More precisely, bordering refers to all those measures — within, outside, and along official state borders — that aim to delineate foreigners’ access to citizenship and membership and to “demarcate categories of people so as to incorporate some and exclude others, in a specific social order” (Guentner et al. 2016, 392). Etienne Balibar (2004, 1) was among the first to pose the idea that external borders have been “reduplicated” in the form of internal borders “wherever the movement of information, people, and things is happening and is controlled.” In this process of de-bordering and re-bordering, borders become an integral part of the daily life of every foreigner, irregular or regular (Agier 2016). This happens either through control technologies that track and trace people's movement (Campesi 2015), or through chauvinist and exclusionary welfare systems where resources are allocated selectively based on the country of origin and nationality of recipients (Dines, Montagna and Ruggiero 2015; Guentner et al. 2016; Patel and Peel 2017; Squire 2015; Yuval-Davis, Wemyss and Cassidy 2018, 2019).

Bordering takes place outside traditional borders through political and economic agreements with migrants’ and asylum-seekers’ origin or transit countries (Frelick, Kysel and Podkul 2018). According to Boswell (2003, 619), this external dimension of bordering has two main characteristics: the “externalization” of migration controls to transit and origin countries and “preventive measures” designed to discourage mobility at the level of individual or societal motivation. The latter involve programs of development assistance to implement economic growth in origin and transit countries and, therefore, prevent people from moving (Latek 2019), while the former includes a number of agreements, such as that between the EU and Turkey in 2016 (Rygiel, Baban and Ilcan 2016), the 2017 Italy–Libya agreement (Montagna 2021), and the funding and construction of camps along the Balkan route to contain the transit of migrants outside EU borders. Both these forms of external bordering has several advantages for the EU and member-state institutions, including less onerous legal and political constraints, little if any accountability in terms of human rights violations, and more effective border control (Frelick, Kysel and Podkul 2018; Hollifield 2000).

Finally, bordering measures take place along land and sea borders (Campesi 2015). Alongside traditional forms of entry control, other controls have emerged that rely on the technologies of biometric identification and surveillance, the use of drones, the deployment of new patrol forces such as Frontex, and the construction of barriers and walls (Amoore 2013; Squire 2015). Although these functions can vary enormously, depending on the tools used, the purpose is the same: keeping unwanted migrants at a distance and filtering their access to the destination country. Biometric screening technologies that allow for the collection of physiological data such as fingerprints, irises, voice timber, and facial features, make it possible to accurately record the characteristics of people arriving into the EU and, thus, to track people's movements (Mountz 2020), including asylum-seekers’ secondary movements (Della Puppa, Montagna and Kofman 2021). For example, Frontex's drones and military equipment used to patrol the Central Mediterranean route, in addition to militarized border practices, monitor movements at sea and allow information to be sent to the coastguard of countries with whom various agreements aimed at controlling migrant flows have been reached (Campesi 2015).

Bordering, as a “tactic of reaction” against people's mobility (De Genova 2017), has been associated with two main processes: the growing securitization of migration and neoliberal globalization. On the one hand, it is part of an increasing global securitization of border controls as a result of the representation of migration as a social (crime) and political (terrorism) danger (Andreas 2003; Bigo 2002) and a challenge to the protection of national identity and welfare provisions (Huysmans 2000). Confinement and securitization policies are, therefore, related to a wider politicization and respond to what Wendy Brown (2014) calls the “desire for walls,” although emphasis on the danger posed by migration often results in increased fear, regardless of the real threat posed by migrants and of border control's actual effectiveness (Yuval-Davis, Wemyss and Cassidy 2019, 7). The security-bordering nexus, which emerged dramatically across the globe after September 2001 and has strengthened in subsequent years (Huysmans 2006; Muller 2012; Winders 2007), has been given a further boost by the use of quarantine ships for arriving migrants, as this article shows.

On the other hand, bordering is a paradoxically constitutive part of and a response to the effects of neoliberal globalization (Yuval-Davis, Wemyss and Cassidy 2019). While the rigid division between core and periphery wanes, global bordering through processes of filtering and differentiating labor power creates new hierarchies of inclusion and exclusion between people and geographical areas (Mezzadra and Neilson 2013, 19). These two nexuses — bordering-security and bordering-neoliberal globalization — are not in opposition but are closely linked. As neoliberal globalization has encouraged a dramatic increase in cross-border mobility (Urry 2007), such mobility has necessitated the creation and use of surveillance systems, both in physical border areas and elsewhere, in a race for border security (Scholte 2005).

Although it is debatable whether travel restrictions alone constitute an effective response to the circulation of the virus, the COVID-19 pandemic has provided new arguments for further bordering (Ambrosini 2020; Spada 2021; Stierl and Dadusc 2022; Tazzioli 2021). The link observed between the rapid circulation of the virus and global integration (Sirkeci and Yucesahin 2020) during the pandemic's first wave, and the racialization of the disease generated by the anti-migrant rhetoric of political elites across the globe (Della Puppa and Perocco 2021; Wihtol de Wenden 2021), have provided national and supra-national authorities with opportunities to tighten already-restrictive migration policies (Ambrosini 2021; Clissold et al. 2020; Elias et al. 2021). The pandemic has also been used to deter mobility through hygienic-sanitary border enforcements in the name of humanitarianism and migrants’ health protection (Tazzioli and Stierl 2021b). As Meer et al. (2020) note, measures that would not otherwise be viewed as legitimate have been taken in the name of public health, in breach of humanitarian laws, some EU member-states ceased asylum procedures, thus stranding asylum-seekers, forced boats carrying refugees out of their territorial waters (Cyprus), and closed their ports and suspended SAR operations at sea (Italy and Malta). While these measures are not “explicit and overt refoulement actions, they have prevented potential asylum seekers from registering at the border” (Meer et al. 2020, 3, see also Della Puppa and Sanò 2021; Guadagno 2020). Far from completely halting people's movement (Tazzioli 2021), these measures have produced a shift from a “hostile Europe” to an “unsafe Europe” (Tazzioli and Stierl 2021a, 2), where migrants’ health, particularly the undocumented or those living in camps and informal settlements, is put at greater risk (Cordova 2021; Guadagno 2020) and the differences between foreign nationals and citizens are exacerbated (Perocco 2021; Roksandic, Mamic and Mikac 2021).

## Doing Research on Migration in Pandemic Times

This article draws on research undertaken as part of an overarching study aimed at understanding migration policy in pandemic times. This study began during the pandemic's first wave. While I was busy reorganizing my teaching and moving all academic-related work online, I was also curious to know more about COVID-19's impact on migrants, their rights, their lives, and their mobility. On the one hand, we could see the racialization of the virus in language — “the Chinese virus” was used by media and politicians alike (e.g., Della Puppa and Perocco 2021; Giacomelli, Musarò and Parmiggiani 2020) — and in attacks on migrants and people from the Far East that took place in different countries, including Italy (Perocco 2021). On the other hand, the right-wing anti-migration parties continued campaigning, creating a narrative that opposed both migrant arrivals and the strict lockdown regulations that Italians were forced to follow, given the impact of these regulations on rights and the economy (Ambrosini 2021). Initially, my aim was to understand whether the Italian government was taking specific and differentiated measures to reduce the impact of the COVID-19 pandemic and lockdown on migrants living and arriving in Italy. It was only after the quarantine ship measure was introduced that the focus shifted, and I started wondering about the rationale of this measure and whether it was intended to protect public health, as stated by the Minister of the Interior, or to create “floating hotspots” aimed at containing mobility. I also wondered if we were seeing something new in migration governance and whether the quarantine ships could be a precedent in EU border control.

To answer these research questions, I selected a sample of 27 key informants from humanitarian and grassroots organizations, lawyers, and news organizations, including radio stations and newspapers, who worked in the migration field. The sample, which I believe is large enough to provide a broad and complete picture of both policy measures and quarantine ships, can be divided into three groups, according to each key informant's affiliation and position. The first group consisted of representatives and practitioners of humanitarian organizations based in Sicily, where most quarantine ships were docked, who were collecting information about the quarantine ships and mobilizing for the transfer of migrants to reception centers on land. The questions I asked them focused on the quarantine ships, whether they visited them or met the migrants who were hosted on the ships. I also asked their opinion about quarantine ships themselves. The second group of informants included activists involved in pro-migrant mobilizations, volunteers and practitioners who worked for humanitarian associations, journalists who wrote about migratory issues, and unionists who campaigned regularly on migrant labor issues. They were chosen for their expertise and knowledge in the fields of migration advocacy and were based in different Italian regions. The questions I asked this group were about government measures to minimize the impact of the pandemic and lockdown on incoming migrants and the foreign population living and working in Italy. Finally, I interviewed seven volunteers from the Italian Red Cross, whose names have been anonymized in the article. The aim was to understand their experiences on the quarantine ships, the positions they covered, how they were recruited and the length of notice before boarding, whether they had any specific training, and other practical issues on life on quarantine ships, including the kind of relationship they established with migrants. Interviews were conducted between the spring of 2020 and early summer of 2021 and ranged in length from 20 to 90 minutes. Given the mobility constraints caused by the pandemic, most interviews were conducted via Zoom.

As can be seen from the initial paragraph of this section, only key informants are involved, and no migrants who underwent the quarantine measure on ships participated in this project. The rationale behind this choice is twofold. First, this study focused on a policy measure and whether it was designed to protect people, both migrants and residents, or borders. In this regard, I also tried to interview officers from the Ministry of the Interior, the head of the Italian Red Cross, and the person in charge of quarantine ships, but they did not respond to my requests. My aim was to contribute to the current debate on the bordering policy measures and practices that characterize current EU migration governance and their application during the pandemic.

Second, when I carried out this study, Italy was in the middle of the pandemic, with continuous lockdowns and mobility restrictions. Circumstances were extremely changeable, and I could not realistically plan any trips across the country to go to the places where quarantine ships were docked or the centers where migrants were held. As documented, researchers must spend a great deal of time in the field and with research subjects to overcome power relations based on different statuses and origins between researchers and migrants and collect meaningful information (Fontanari 2017; Montagna 2018; Semi 2010). The need to spend time in the field is especially true when research subjects are migrants who arrive irregularly and whose journeys are characterized by traumatic experiences, with their current status uncertain and under the control of government officials. Such migrants are often traumatized and legitimately suspicious of the interviewer and their role (Anderson, Rogaly and Ruhs 2012; Düvell, Triandafyllidou and Vollmer 2008; Sanchez-Ayala 2012; van Liempt and Bilger 2009, 2012; Vargas-Silva 2012). Since the conditions for reducing the distance and neutralizing power relations with migrants were lacking due to the pandemic and the resulting mobility restrictions, I decided not to include them in fieldwork and to rely exclusively on key informants, who could be reached more easily, both because I knew them personally from having participated in other research projects and because their names were publicly available online and elsewhere.

## Normative Framework of Quarantine Ships

As the data show, provisions aimed at mitigating, and eventually interrupting, COVID-19's spread among people resident in Italy proliferated during the pandemic's first wave, when the measure of quarantine ships was introduced. Between January and April 2020, a total of 246 different COVID-19-related measures were issued by the Italian government, an average of 61.5 per month, including 103 in March alone.^3^ Among this array of measures passed with the objective of reducing social contact and the circulation of the virus, only two specifically concerned migrants (Associazione per gli Studi Giuridici sull'Immigrazione 2021). On April 7, 2020, a decree adopted by the Minister of Infrastructure and Transport, in agreement with the Minister of Foreign Affairs and International Cooperation, the Minister of the Interior, and the Minister of Health, established that by virtue of the Hamburg Convention,^4^ “for the entire duration of the national health emergency deriving from the spread of the COVID-19 virus, Italian ports do not meet the necessary requirements for the classification and definition of ‘place of safety’ for rescue cases carried out by naval units flying a foreign flag (i.e., SAR ships) outside the Italian SAR area” (Avvenire 2020). In other words, by equating Italian ports with unsafe places — which, in the Hamburg convention, are usually either countries at war or where respect for human rights is not guaranteed — people rescued at sea were not allowed to disembark, “evading the mandatory constitutional and international obligations regarding the right of asylum, protection from the risk of undergoing inhuman and degrading treatment, and SAR at sea” (Associazione per gli Studi Giuridici sull'Immigrazione 2021, 2). The rationale behind this decree was the alleged impossibility of ensuring necessary health care and basic services, due to the pandemic, and the risk that migrants’ arrival by sea would strain the national health service (Camera dei Deputati 2021). But the decree was also in line with the security decree imposed by Salvini, the then Minister of Interior and leader of the far-right *League* party, in 2019 to limit the SAR activities of NGO ships engaged in the central Mediterranean (Vassallo Paleologo 2021).

On 12 April, Civil Protection, the Italian government department in charge of emergencies including pandemics, arranged for the quarantine of all migrants rescued at sea or who arrived after autonomous landings either on ships or in land accommodation (Ambrosini 2021). It decreed (Dipartimento della Protezione Civile 2020):

With reference to *people rescued at sea* and for whom it is not possible to indicate the ‘place of safety’ (*porto sicuro*), the implementing body, in compliance with the protocols shared with the Ministry of Health, can use *ships* to carry out the period of health surveillance… With regard to *migrants arriving on the national territory independently*, the implementing body identifies, after consulting the competent Regions and local health authorities, through the competent prefectures, *other areas or structures* to be used as accommodation for the period of health surveillance… In the event that it is not possible to identify the aforementioned structures on the territory, the implementing body arranges for the accommodation of migrants for the purposes of fiduciary and quarantine isolation also on *the aforementioned ships* (My italics).

The two decrees mentioned above are complementary. While the first declared that Italy was no longer a “place of safety” for migrant disembarkation, the second provided an alternative for people rescued in Italian territorial waters, instituting quarantine ships to which they could be safely transferred. Enforcement started straight after the second decree was passed (Camilli 2020). On April 13, 2020, after being denied safe harbor for two weeks, 183 refugees, including 33 minors and two women, were granted permission to disembark from the SAR ships, *Alan Kurdi* and *Aita Mari*, and immediately transferred to the cruise ferry, *Rubattino*, about a mile from the port of Palermo, where they were quarantined for nearly 20 days (ibid.). In May 2020, the *Rubattino* was replaced by the *Moby Zazà*, and other ships were added over the summer, due to the increasing number of arrivals, the lack of room at the Lampedusa hotspot, and protests, often led by anti-migrant parties, against COVID-19 centers for migrants (Ziniti 2020). The scope of quarantine ships also expanded: contrary to the provisions of the 12 April decree, the ships began to be used for quarantining those rescued from Italian-flagged ships and those disembarking independently, by then the majority of arrivals, who were initially turned over to “onshore” facilities for the period of health surveillance (Associazione per gli Studi Giuridici sull'Immigrazione 2021). By early Autumn 2020, a small fleet of up to seven ships had been established, each of which could host between 400 and 900 migrant guests and cost between 1 and 2 million euros per month, depending on the number of people housed (MicroMega 2021, 9 November 2021).

## The Securitarian Shifting of Quarantine Ships

Because of the actors involved, especially the Italian Red Cross, and the mission's aim to protect local communities’ and arriving migrants’ health, quarantine ships can be seen as humanitarian interventions. Humanitarian interventions form what Fassin (2011) calls “humanitarianism,” the third pillar of contemporary governmentality, along with policing and economics. While combining humanitarian principles, such as safeguarding life and alleviating suffering, these interventions are not without ambiguity (ibid.). In bringing relief to suffering people, humanitarianism can also reproduce inequality and subordination, and the entities promoting it can be confronted by violence (Agier 2016; Fassin 2011; Fontanari 2018; Montagna and Grazioli 2019; Tazzioli 2016). “Humanitarian reason” (Dines 2018) and its actors, however genuinely motivated, may, therefore, participate in people's confinement (Campesi 2015; Dines 2018), turning humanitarianism into a smokescreen in the name of higher principles that alleviate crises within a securitizing logic (Tazzioli 2016).

It is within a humanitarian framework that the Italian government signed a protocol with the Italian Red Cross, one of the world's leading humanitarian organizations, for the management of health activities, social and psychological support for migrants and volunteers, logistics, and, at a later stage, legal assistance on board the quarantine ships (MicroMega 2021, November 9, 2021). The Red Cross's engagement with the Italian government in humanitarian bordering practices dates to the late 1990s, when the Italian Red Cross was charged with managing the country's migrant reception system; more recently, it has been involved in the management of hotspots (Tazzioli 2016). Although the Red Cross has often been accused of privileging government demands over protecting migrants and of ignoring episodes of violence and mistreatment (Sciurba 2008; Tazzioli and Garelli 2018), it emerged as the most qualified to manage complex facilities such as quarantine ships housing several hundred migrant guests, given its experience in managing crisis situations, its network of local and regional offices, and its group of around 150,000 volunteers and 100 employees specialized in health and social assistance.

According to the protocol signed by the Red Cross, employees and volunteers were expected to provide healthcare, advice, and psychological support through counselling to arriving migrants and to identify physically and psychologically vulnerable migrants (Associazione per gli Studi Giuridici sull'Immigrazione 2021: 5). Interviews with informants, however, revealed that despite efforts to interpret the humanitarian mandate and meet migrants’ needs while on board the ships, much of the Red Cross volunteers’ support fell short of the mission’s goals. From these interviews, it emerged that volunteers were often untrained, with little or no instruction about their tasks when they were recruited, while many did not know that much about quarantine ships was improvised:

I knew nothing about quarantine ships and had never heard of them before that message. If I must tell the truth, I still didn’t understand what they were until I got on them, not even a faint idea of what they were. When I asked my local office, they did not know how to give more information than those few notes that were written in that Telegram message, and, therefore, I really had absolutely no idea if I was going on a tub or on a cruise ship… They promised some training, but it never came (Marco, Red Cross).

Similarly, another volunteer was called by the coordinator of the local group of the Italian Red Cross one evening in late summer 2021 and told that her plane to Catania left the following day. When she asked for more details about clothing, she was told to plan “as if you were going on holiday” (MicroMega 2021, November 9, 2021). With no preparation course, no psychological interviews, and no tests, a few hours after she received the call, she found herself on a bridge with hundreds of people crammed together, where “you couldn’t tell who was positive, who was negative and who had had close contact with the positives” (ibid.).

The number of volunteers on board the ships was insufficient to provide support on a vessel that could accommodate up to 800 people (Vita 2020). Due to the length of shifts and the number of migrant guests, those who were trained and had the skills to deal with the issues raised by vulnerable subjects were often engaged in other tasks:

We did everything, the welcome on arrival, the preparation of the welcome kit with the clothes, the soap, and everything they need to wash, 1,000 welcome kits to give each person at each boarding, the logistics and, therefore, the organization of the warehouse, the distribution of meals at breakfast, lunch, dinner. Then, I did the COVID shift, that is, the shift on the bridges completely dedicated to the COVID positives to check if they needed anything and, of course, the normal shift on the bridges where I had to take care of all the needs they might have, from toothache to requests for food or blankets. We made sure everyone was comfortable and at the same time complied with anti-COVID rules (Alessandro, Red Cross.)

If one of the aims onboard the ships was to identify vulnerable cases, the shortage of professionals and of mediators who spoke the same language as the migrant concerned meant that this aim was hardly possible. As a result, very few migrant guests were identified as vulnerable, and one of the humanitarian tasks was lost (MicroMega, November 9, 2021).

On the other hand, because there were no police on board the ships and because professional security was hired to avoid damage to the ships’ structure, volunteers were responsible for internal security and for enforcing the rules:

We had to ensure that the migrants kept their distance, wore masks, did not change decks, as they were in quarantine, they could not pass from one bridge to another. We had to cool down the situation when there were moments of tension, accompany them on the outside deck when they wanted to smoke a cigarette to prevent them from jumping off the ship, or to intervene when, for example, they try to self-harm (Alessandro, Red Cross).

Most importantly, with regard to the ships’ securitization and transformation into “floating hotspots” and on the basis of interviews, volunteers, in collaboration and coordination with border authorities, also played an active role in identifying those who arrived and, thus, in filtering migrants into those who could apply for asylum and those who could not. Volunteers acted as “immigration officers” while on board as humanitarian workers. They verified new arrivals’ identity, registered them, and communicated these data to the Ministry of the Interior: “Everyday, hours and hours of calls with the Ministry of the Interior to see that all the people were registered and then the same thing with the disembarkation, check-out for all the people… There was really everything to do” (Alessandro, Red Cross). Their role was, therefore, crucial in the management of borders, as any decision regarding application for asylum or return migrants who were considered ineligible for protection depended on this information.

Recalling certain ambivalences of humanitarian interventions *vis-à-vis* migratory phenomena (Dines 2018; Fassin 2011), particularly shifts between contributing to border security and safeguarding migrants’ health, two types of volunteers can be identified among Red Cross volunteers. These different types are constructed according to how they interpreted their roles on the quarantine ships and how they saw their relationship with the migrants to whom they were providing relief. The first type is the enthusiast. For them, volunteering was a compassionate mission which relied on constructing the migrant as the “needy other” with little or no agency or individuality through the opposition between a generic “us” and a generic “them”: “Things [the volunteer was referring to clothes and care] that for us are normal for them are enormous” (Lucia, Red Cross). This representation replaced one constructed before volunteers’ experience on the ships:

I didn’t know anything. I went there out of curiosity because I wanted to understand what was… because you know very well that you hear about every kind of thing these migrants who arrive, that they don’t need anything, that they have a mobile phone and that… unfortunately, it is not exactly like that (Marta, Red Cross).

As emerged from my interviews with informants, for some of them confining migrants was a necessary and unproblematic way to protect public health. Volunteering for the Red Cross was not only *instrumental*, a means to an end where the end was the humanitarian support of suffering persons, but also *ideological*, as volunteers identified themselves with the Red Cross’ seven principles of humanity, impartiality, neutrality, independence, volunteerism, unity, and universality. Their view of the intervention as reflecting the Red Cross’ principles deterred them from questioning the transformation of their position into a security role and the quarantine ships’ bordering function. The volunteers of this first type did their best to cover the organizational gaps and the lack of adequate resources required to carry out a humanitarian mission. They did so in the name of the Red Cross's principles, to which they adhere. At the same time, they did not interrogate the meaning of their action as Red Cross volunteers and the role of the quarantine ships in the COVID-19 emergency.

The second type of volunteers is the disillusioned. For this group, volunteering on the quarantine ships came up against the operation's organizational and purely humanitarian limitations. They reported feeling in a bubble, totally overwhelmed by the tasks they had to do and by migrants’ constant demands, with no chance to stop and think. They were skeptical about a system that they identified more as a bordering measure than a health measure. For them, initial enthusiasm gave way to frustration at not being able to cope with the problems and expectations of migrants on the ships, and “you realize that the ships don’t solve anything; they don’t even guarantee a true quarantine” (Martina, Red Cross). It is among this type that the idea emerged that “the whole system is not designed for the welfare of migrants but to reassure Italian public opinion that the ‘migration problem’ is under control” (ibid.). My interviews and those given to the media indicate that these volunteers felt constrained in their roles as humanitarian workers whose main function was to safeguard borders, rather than to provide aid to people in need. Despite widespread skepticism, many volunteers agreed to do more than one operation and stayed for several months, with the idea that their individual efforts could overcome the issues related to the mission: “We can still make a difference” (Alessandro, Red Cross).

This section has shown the ambivalence of Red Cross intervention, which combined a weak humanitarianism, as it was unable to meet the needs of migrants housed on quarantine ships, especially the vulnerable, with security practices. This ambivalence is reflected in the activities of Red Cross volunteers, who oscillated between health and social support and security and policing practices such as preventing migrants from getting involved in fights or collecting migrants’ identity data for the Ministry of the Interior. The tension between humanitarianism and security emerged in the words of the most skeptical volunteers, who saw the criticality of their role, and shows how even a humanitarian intervention such as the one they were involved in can become complicit in bordering politics. The next section examines the impact of this double standard on migrants confined on the quarantine ships.

## Confined in a Suspended Temporality

The humanitarian quarantine operation started with initial photographic identification, fingerprinting, and COVID-19 testing of migrants who were rescued at sea or had arrived independently (Spada 2021). As indicated by my interviews and by some volunteers’ interviews with the media, boarding the quarantine period was immediate, regardless of whether migrants tested positive. Each ship had five to seven decks, one of which was for COVID-19 positives and the top deck of which was for the around 25 Red Cross operators. There was no clear separation or distribution between nationalities, with the consequence of frequent fights among migrants. Moreover, while families were kept together wherever possible, women were often forced to stay on the same decks as men, even when they requested separate decks. This lack of separation meant that trafficked women may have traveled with their exploiters, resulting in cases of prostitution or abuse on board (MicroMega 2021, 9 November 2021). Once embarkation was completed, the ships were anchored outside the ports of Lampedusa, Palermo, Trapani, Augusta, Porto Empedocle, and Bari and returned to port only to embark or disembark other people. The period of stay on the ships varied between 2020 and 2021: until spring 2021, negative cases had to stay for a minimum of 15 days. After spring 2021, the period of stay was reduced to 10 days, in accordance with measures for the rest of the Italian population; migrants who tested positive stayed until they tested negative. There were frequent cases of people having to wait up to a month before testing negative.

Contrary to what Red Cross officials said,^5^ when migrants boarded the quarantine ships, they had little awareness of what was happening. They learned, with surprise, that, after being rescued or landing on Italian shores, they would be put on a quarantine ship (Associazione per gli Studi Giuridici sull'Immigrazione 2021). Only once on board were they informed by the operators, without any formal written communication, that for health reasons related to COVID-19's spread, they had to stay in isolation, without any contact with the outside world and without being able to disembark, thus being deprived of their personal freedom, for an indefinite number of days (Giacomelli 2020). In the operation's first few months,

Many didn’t even know what COVID was or what quarantine was, as we did in the first weeks of the pandemic. They didn’t know what part of Italy they were in, so we attached maps to explain exactly where we were. We immediately explained to them what the Red Cross is and why we were there, what quarantine is, what it means to be positive, and what happens if you become positive. However, yes, they had no idea what was going on. We had to tell them everything upon boarding (Andrea, Red Cross).

As evidenced by my findings, on board, migrants’ temporality was mostly characterized by waiting and inactivity, existential suspension and legal uncertainty, since only very limited information was given to them about what would happen after the quarantine period. Agier (2010, 72) writes about migrant camps as “models of uncertainty” where “spaces and populations [are] administered in modes of emergency and exception, where time seems to have stopped for an indeterminate period”; and this reasoning also applied to quarantine ships, which became “space[s] of exception” where people lived an uncertain suspended temporality.

During this enforced suspended time, there were no organized activities for adult guests, such as language courses, or children: “any initiative depends on the individual operator, who might make themselves available to play a bit with some children” (Laura, Red Cross). Most of the day was spent chatting with other migrants or with Red Cross volunteers, but always in the corridors on their deck; for safety reasons, access to the outside decks was allowed for about half an hour a day and always under the escort of volunteers. No formal information was given to migrant guests about their test results, where they were going to disembark, or what was going to happen after the quarantine period. Medical assistance and even psychological support, particularly for vulnerable migrants, were reported as insufficient for migrants’ problems.

Legal assistance was only introduced at a later stage, following protests by humanitarian organizations (MicroMega 2021). Before its formal introduction, legal assistance was provided by volunteers who were not trained for the task and only on a voluntary basis, as the Red Cross asked volunteers not to provide this kind of information (Associazione per gli Studi Giuridici sull'Immigrazione 2021). However, one of the most important tasks of legal aid, the collection of asylum applications and their handover to the authorities, was soon taken away from the legal workers on the ships (MicroMega 2021). As the local offices of the Ministry of the Interior were quickly inundated with asylum applications, to discourage asylum-seekers from applying, it became “the police who collect them in port, with cultural mediators who often play the game of the Italian institutions and say, ‘sign here’, without explaining that this is the form renouncing the asylum application” (MicroMega 2021). Quarantining became a way for the Ministry of the Interior to gain time and organize expulsions and repatriations to those countries with which Italy had an agreement, like Tunisia, or that were considered safe, such as Algeria, Egypt, and Morocco.

Cases of self-harm among migrants, as well as attempts to escape by jumping from ships, were frequent and sometimes lethal, as in the case of Bilel Ben Masoud, a 22-year-old Tunisian man who threw himself into the sea from the *Moby Zazà* on May 20, 2020 and died (Gianguzza and Karkouri 2020). Although communication between migrants and the outside world was difficult, as the ships did not have wi-fi and migrants could only rely on their own means or those provided by volunteers, “they [migrant guests] had received messages from their friends and knew it was easier to escape than from there [quarantine ships]” (Paolo, Red Cross).

In this context of social and physical isolation, revolt became a way of expressing requests, being heard, or negotiating conditions on quarantine ships (Gianguzza and Karkouri 2020). On October 10, 2020, a riot broke out on the quarantine ship, *Rhapsody*, during a long disembarkation in Bari, after rumors circulated that remaining migrants would be taken to Lampedusa and deported to Tunisia (La Gazzetta del Mezzogiorno 2020). On August 26, 2021, a group of migrants arriving in Augusta from Lampedusa protested quarantine; some jumped overboard, others vandalized the ship, and some tried to escape but were blocked by police on the quay (La Repubblica 2021). Other scuffles happened over this period that went unreported in the media. During such events, volunteers were mandated to lock themselves in cabins until the incidents were over, although they were never the protesters’ target (MicroMega 2021, November 9, 2021) and their security role ceased:

What happens outside is of no concern to the volunteers, and security on board is only concerned with the safety of the bridge. So the decks are abandoned, you wait a few hours, until the announcement comes that everyone can leave and get back to work (Massimo, Red Cross).

As evidenced by my interviews with Italian Red Cross volunteers, after the quarantine period, migrants were taken to the mainland. Migrants returned their badges to the Red Cross personnel and were handed over to the police, which sorted them according to their right to apply for asylum, usually determined by nationality rather than by actual risk of persecution. Depending on whether they could apply for asylum, some migrants were taken to reception centers, while others, mostly from North Africa, were transferred to detention centers to await deportation to their origin country. Others still were given a decree of expulsion stating they had seven days to leave Italy without any hearing or chance of appeal against it.

## Concluding Remarks: Health Measure or “Floating Hotspots”?

This article aims to contribute to the debate on bordering and, more specifically, to the developing work on how the pandemic has been used for further border closures in several areas across the globe including the Mediterranean (Stierl and Dadusc 2022). As scholars have noted, these policies have taken different forms, from applying differential blocking measures for citizens and migrants (Tazzioli and Stierl 2021b) to closing ports to SAR operations at sea (Guadagno 2020), from preventing potential asylum seekers registering at borders (Meer et al. 2020) to scaling back assistance within humanitarian spaces (Sanò and Tabar 2021). The pandemic has therefore provided an "excuse" (Stierl and Dadusc 2022) for refining repressive logic and practices that reinforce internal and external borders in violation of human rights (Spada 2021).

With reference to this body of scholarship and to the broader literature on border and security, and with a specific focus on the Mediterranean, my article has looked at one device of the several used to control international migration during the COVID-19 emergency. We have seen how quarantine ships designed to address the COVID health emergency became “floating hotspots” whose aim was to filter arriving migrants in Italy through the Central Mediterranean route. This humanitarian measure aimed at ensuring the safety of migrants and natives was turned into a securitarian device enriching migration policies with new border control tools. Such use of quarantine ships as “floating hotspots” was highlighted by several informants, including Red Cross volunteers and members of humanitarian NGOs. They emphasized how, once quarantine was over, the border authorities delivered the decrees of expulsion directly, without any preliminary investigation into whether the migrants temporarily confined on the ships had the right to apply for asylum and only on the basis of nationality (Spada 2021). As for the hotspot approach launched by the 2015 European agenda on migration, quarantine ships were spaces of temporary confinement while decisions were made on whether arriving migrants were eligible to apply for asylum or should be deported before their applications were processed.

The case of Tunisian citizens, who were subject to the systematic use of return procedures, is emblematic: from disembarkation to the Lampedusa hotspot to the quarantine ship to the *Centri per il Rimpatrio* (Repatriation Centres), Tunisian migrants faced a near-automatic pipeline, which was consolidated during nearly two years of the quarantine ship implementation (Gianguzza and Karkouri 2020). All other arriving migrants were given a decree of deportation, although they were not physically deported back to their origin country.^6^ Apart from with Tunisia, Italy has virtually no agreements with most migrants’ origin countries for their readmission (D'Angelo et al. 2017).

The COVID emergency provided the context for the introduction of the norm that an initial filtering of arriving migrants could be conducted at sea under a kind of “administrative detention” (i.e., a restriction on freedom of movement without trial, albeit for a limited period and for alleged public health reasons, but without criminal prosecution) (Vassallo Paleologo 2021). This idea — which, as we saw in the introduction, was raised and then abandoned during the most acute period of the humanitarian crisis in the Mediterranean between 2014 and 2017 — was finally put into practice (Spada 2021, 54). In line with the pre-existing “hotspot approach” of the EU migration agenda, the quarantine ships made possible the establishment of a precedent for the institutionalization of “floating hotspots,” despite the proven violations of both personal freedom and fundamental rights to asylum, information and health.

## Footnotes

1 Grandi Navi Veloci is an Italian shipping company, based in Genoa, that operates ferries between mainland Italy, Sicily, Sardinia, France, Albania, Morocco, and Tunisia.

2 The idea of using quarantine ships or floating hotspots to “carry out identification operations directly on board, without letting anyone escape” (*la Repubblica*, May 18, 2016) was suggested by the Italian government in May 2016 to better control who was arriving. After initial support from both the EU and UNHCR, both quarantine ships and floating hotspots were ruled out because they were not deemed to be in line with the principles of access to the asylum procedure for those requesting it. See the answer by the European Commissioner for Migration, Home Affairs and Citizenship, Dimitris Avramopoulos, 10 August 2016, to the question by Nicola Caputo MEP from the Socialists & Democrats group: *Answer given by Mr. Avramopoulos on behalf of the Commission* available at https://www.europarl.europa.eu/doceo/document/P-8-2016-004213-ASW_EN.html.

3 For more information on the provision during the emergency, see https://www.openpolis.it/coronavirus-lelenco-completo-degli-atti/, accessed April 9, 2021.

4 The *International Convention on Maritime Search and Rescue (SAR)*, adopted at a Conference in Hamburg in 1979 and entered into force in 1985, was aimed at developing an international SAR plan so that, no matter where an accident occurs, the rescue of persons in distress at sea will be co-ordinated by a SAR organization and, when necessary, by co-operation between neighbouring SAR organizations (International Convention on Maritime Search and Rescue, available at: https://www.imo.org/en/About/Conventions/Pages/International-Convention-on-Maritime-Search-and-Rescue-(SAR).aspx). See also Papanicolopulu (2016).

5 In an interview, Francesca Basile, Head of the Migration Organisational Unit at the Italian Red Cross, said: “We explain things in a transparent way, with all the details, for example how the first and second swabs are performed. We explain that those two weeks have a health reason for their own good and for the communities that will then welcome them” (Vita, 8 September 2020).

6 In 2021, when more than 60,000 migrants arrived in Italy, only 3,420 were deported to their origin countries. Of these, 57 percent were Tunisians, 19 percent Albanians, and 8 percent Egyptians, while the remaining 16 percent were from other countries (Garante Nazionale dei diritti delle persone private della libertà personale 2022).
